# Gender Differences at the Onset of Autoimmune Thyroid Diseases in Children and Adolescents

**DOI:** 10.3389/fendo.2020.00229

**Published:** 2020-04-17

**Authors:** Valeria Calcaterra, Rossella E. Nappi, Corrado Regalbuto, Annalisa De Silvestri, Antonino Incardona, Rossella Amariti, Francesco Bassanese, Andrea Martina Clemente, Federica Vinci, Riccardo Albertini, Daniela Larizza

**Affiliations:** ^1^Pediatric and Adolescent Unit, Department of Internal Medicine and Therapeutics, University of Pavia, Pavia, Italy; ^2^Pediatric Endocrinology Unit, Department of Maternal and Children's Health, Fondazione IRCCS Policlinico San Matteo, Pavia, Italy; ^3^Research Center for Reproductive Medicine, Gynecological Endocrinology and Menopause, Fondazione IRCCS Policlinico San Matteo, Pavia, Italy; ^4^Department of Clinical, Surgical, Diagnostic and Padiatric Sciences, University of Pavia, Pavia, Italy; ^5^Biometry and Clinical Epidemiology, Scientific Direction, Fondazione IRCCS Policlinico San Matteo, Pavia, Italy; ^6^Laboratory of Clinical Chemistry, Fondazione IRCCS Policlinico San Matteo, Pavia, Italy

**Keywords:** gender difference, autoimmune thyroid disease, children, adolescents, thyroiditis

## Abstract

**Background:** The incidence of autoimmune thyroid diseases (ATD) may vary with the beginning of reproductive function, although few reports differentiate the incidence before and during the onset of puberty, examining gender bias. We analyzed onset of ATD in a pediatric population to assess gender differences in onset age, disease subtype, pubertal status, autoimmune co-morbidity, family history and treatment, focusing on the interaction between gender and pubertal stage.

**Patients and methods:** We retrospectively recorded 382 children and adolescents with ATD. In each patient physical examination was considered. The presence of other associated autoimmune diseases (AAD) and familial predisposition was also recorded.

**Results:** Predominant prevalence was noted in females compared to males (*p* < 0.001), both in Hashimoto's diseases (HD or HT) and Graves' disease (GD) (*p* < 0.001). Mean age at diagnosis showed no significant difference between sexes (*p* > 0.05). A higher prevalence in pubertal subjects was noted compared to prepubertal (*p* < 0.001, particularly HT in early and GD in late pubertal stage), without sexes difference intra-(prepubertal vs. pubertal) and inter-puberty groups (prepubertal vs. early pubertal vs. late pubertal). Both in HT and in GD, the prevalence of autoimmune associated diseases (AAD) was higher in males compared to females (*p* = 0.04), with similar distribution according to the pubertal maturation. The familial predisposition was similarly distributed in both genders (*p* > 0.05) and into pubertal stages (*p* > 0.05).

**Conclusions:** Females are more prone to develop ATD during puberty, earlier in HT than in GD. The effect of puberty is not different between genders, suggesting the role of additional factors other than hormones. The screening for detection of ATD is recommended in all patients with positive family history and other autoimmune diseases, mostly in males. Considerations of gender in pediatrics could be important to define pathogenic mechanisms of ATD and to help in early diagnosis and clinical management.

## Introduction

Autoimmune thyroid diseases (ATD), which includes both Hashimoto's thyroiditis (HT) and Graves' disease (GD), are the most common etiology of acquired thyroid dysfunction in pediatrics (1–3) ATD are characterized by the production of anti-thyroid antibodies, by an infiltration of autoreactive B and T lymphocytes into the thyroid parenchyma and by alterations in thyroid function (hyperthyroidism in GD, normal function or subclinical/overt hypothyroidism in HT) (4).

Both polyautoimmunity and familial autoimmunity are typical findings of ATD (5, 6), supporting that the risk of developing disease is related to genetic susceptibility, with environmental factors playing a role in triggering disease in susceptible individuals (7). Different genetic factors are associated not only with susceptibility to a certain disease, but also with specific autoantibodies and disease phenotypes (7).

In pediatrics, ATD usually occur in puberty (1, 2) but they may occur at any time, rarely even in children under 1 year of age (2). The incidence of autoimmune disease may vary with the onset of reproductive function, although the incidence before and during the onset of puberty. In particular, the consideration of gender exist in examining the data in pediatric endocrinology, whereas a higher incidence rate of ATD in the adult female population is well-described (7). Reasons underlying a higher rate of ATD diagnosis in women are unclear. Moreover, we lack a real knowledge on gender-specific phenotypes.

That being so, we analyzed a pediatric population with onset of ATD to assess the gender differences as regard onset age, disease subtype, pubertal status, autoimmune co-morbidity, family history and treatment, focusing on the interaction between gender and pubertal stage. The gender specific characteristics of disease across puberty may help early diagnosis and clinical management.

## Methods

### Patients

We retrospectively recorded 382 children and adolescents with ATD (HT and GD) who have been referred to the Pediatric Endocrinology and Diabetology Unit of the Fondazione IRCCS Policlinico San Matteo for diagnosis and treatment over a decade (2009–2019).

ATD diagnosis was based on the finding of one or more positive thyroid autoantibodies and a characteristic thyroid ultrasound, lacking homogeneity, with a hypogenic or mixed echopattern.

In each patient, physical examination was performed. The presence of eventual associated autoimmune diseases (AAD) at the ATD diagnosis was also recorded.

Positive family history of autoimmune thyroid disorders was collected (only first-degree relatives were considered significant) in order to analyze the effect of familial predisposition on the development of ATD.

The protocol was performed with the approval of the Ethical Committee of Fondazione IRCCS Policlinico San Matteo, Pavia, Italy and according to the Declaration of Helsinki. All participants or their responsible guardians were asked their written consent after being informed about the nature of the study.

### Physical Examination

Physical examination of patients at first diagnosis included evaluation of height, weight, BMI (calculated as body weight in kilograms divided by height in meters squared), stages of puberty according to Marshall and Tanner (8, 9).

Anthropometric and blood pressure measurements were performed as previously described (10).

Pubertal development was classified as: prepubertal = Tanner 1; early puberty = Tanner 2–3; late puberty = Tanner 4–5.

### Biochemical Data

TSH, FT4 and FT3 and anti-thyroid peroxidase antibodies (TPOAb), anti-thyroglobulin antibodies (TGAb) anti-thyrotropin receptor antibodies (TRAb), were measured as previously reported (11).

Thyroid ultrasound studies were performed by the same operator (VC) with patients in a supine position with the neck hyperextended, by using an Aloka machine (Aloka Prosound α5, Aloka, Tokyo, Japan) with a 7–13 MHz linear transducer.

### Statistical Analysis

Qualitative variables were described as counts and percentage and the chi square test was used to compare the differences between groups. The Shapiro-Wilk test was used to test the normal distribution of quantitative variables. As quantitative variables were normally distributed, the results were expressed as the mean value and standard deviation (SD) and compared between groups with *t*-test.

## Results

### Overall Population With ATD

Out of the 382 patients with ATD (361 HT and 21 GD) included in the study, 325 (85%) were females and 57 (15%) were males (*p* < 0.001). Mean age at the diagnosis was 11.58±0.21 years without significant sexes difference (females 11.55 ± 0.23 vs. males 11.73 ± 0.47, *p* = 0.7).

**Figure 1:**
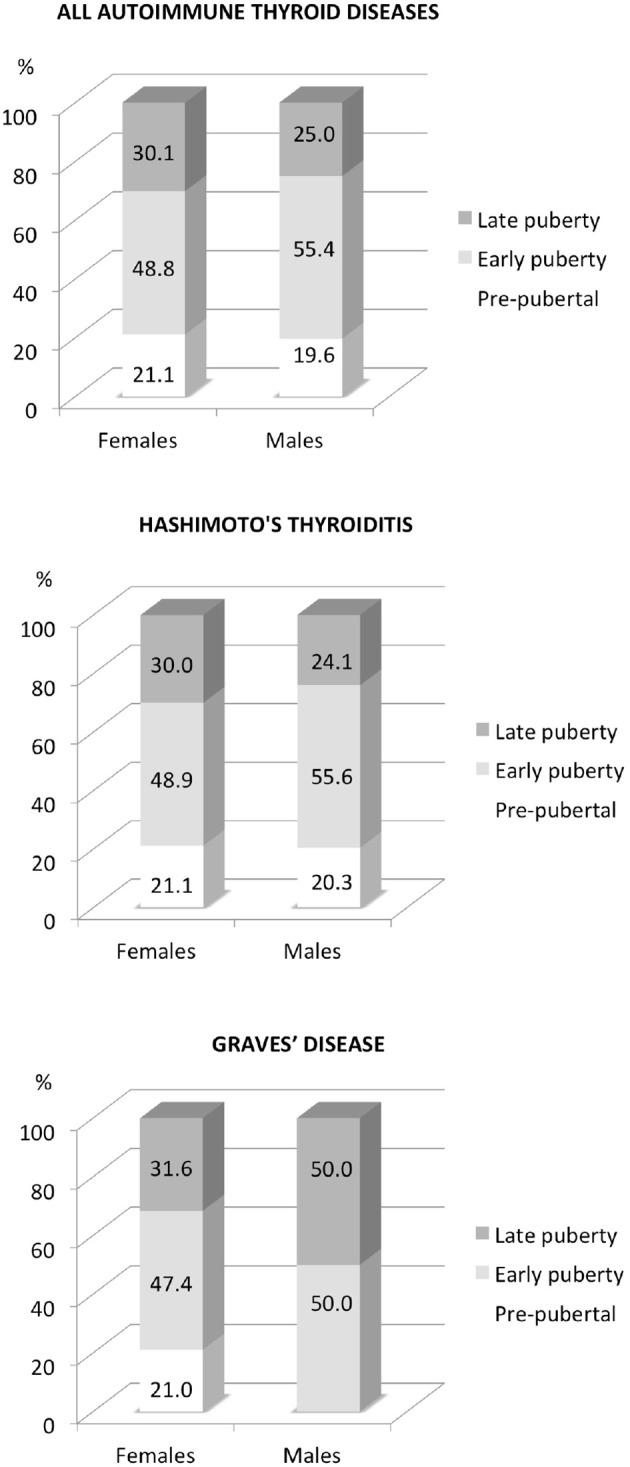
Sex distribution of autoimmune thyroid diseases according to pubertal stages.

Pre-pubertal status was present in 79 (20.9%) of our patients, and pubertal condition in 298 (78%), *p* < 0.001; in particular early puberty was recorded in 188 (49.74%) and late puberty in 111 (29.37%), *p* < 0.001, without sexes difference intra- (prepubertal vs. pubertal *p* = 0.6) and inter-puberty groups (prepubertal vs. early pubertal vs. late pubertal, *p* = 0.86), Figure 1.

AAD was noted in 100 (26.18%) of our overall population, with higher prevalence in males compared to females (37.0% vs. 24.31%, *p* = 0.04). Mean age of the subjects with poliautoimmunity was similar compared to patients with a single disease (11.45 ± 0.24 vs. 11.93 ± 0.81, *p* = 0.8) and there was no difference between gender or according to pubertal stage distribution (*p* > 0.05). Celiac disease was detected in 58 of subjects (58%; 47F/11M, *p* < 0.01 and in 2 cases type 1 diabetes was also present), type 1 diabetes in 19 (19%; 13F/6M, *p* < 0.01), autoimmune gastritis 6 (6%; 3M/3F, *p* < 0.01), vitiligo 11 (11%; 9F/2M, *p* < 0.01), and alopecia in 9 (9%; 8F/1M, *p* < 0.01) children.

Positive family history of autoimmune diseases was reported in 204 of our patients (53.83%), without difference in prevalence between males and females (*p* = 0.7). Mean age at onset was not different in patients with or without a positive family history (11.77±0.26 vs. 11.29 ± 0.35, respectively, *p* = 0.3), as well as the gender lineage did not make any difference (*p* = 0.25). Distribution of the pubertal maturation in the groups with or without positive family history was similar taking into account the gender bias (*p* = 0.21 and *p* = 0.84, respectively).

At the onset of disease, hormonal treatment was started in 204 (53.4%; 183 with L-thyroxine and 21 with metimazole) of children and the rate was similar in males and females (*p* = 0.8) with no gender difference also according to pre- and pubertal condition (*p* = 0.6 and *p* = 0.8, respectively).

### Hashimoto's Thyroiditis (HT)

Out of the 382 patients with ATD, 361 subjects were diagnosed with HT, with predominance of female population (*F* = 306, 84.7% vs. M = 55, 15.2%, *p* < 0.001). Mean age at diagnosis was 11.58 ± 0.21 years without significant difference between gender (females 11.55 ± 0.23 vs. males 11.73±0.47, *p* = 0.7).

Pre-pubertal status was detected in 21.3% of our patients, and pubertal condition in 78.7%, *p* < 0.001; in particular, early puberty was recorded in 50.57% and late puberty in 28.14%, *p* < 0.001, without difference between gender intra- (prepubertal vs. pubertal, *p* = 0.61) and inter-puberty groups (prepubertal vs. early pubertal vs. late pubertal, *p* = 0.9), Figure 1.

AAD was diagnosed in 27.14% of the study sample with ATD, with higher prevalence in males compared to females (36.84% vs. 19.38%, *p* < 0.01). The mean age of the patients with poliautoimmunity was similar in comparison with those carrying only one disease (11.45 ± 0.24 vs. 11.93 ± 0.81, *p* = 0.8); no difference between gender was found (*p* = 0.9). Celiac disease was detected in 56 of subjects (56%; 47F/9M, *p* < 0.01), type 1 diabetes mellitus in 17 (17%; 13F/4M, *p* <0.01), autoimmune gastritis in 6 (3F/3M; 6%; *p* < 0.01), vitiligo in 11 (11.2%; 8F/2M, *p* < 0.01), and alopecia in 9 (9.1%; 8F/1M, *p* < 0.01) children. Distribution of the pubertal stages in the patients with or without associated autoimmune diseases was not different according to the gender (*p* = 0.9 and *p* = 0.4, respectively).

Positive familiarity for autoimmune diseases was reported in 54.47% of our patients, without a different prevalence between males and females (*p* = 0.7). Mean age at onset in subjects with positive familiarity was not different compared to patients without (11.77 ± 0.26 vs. 11.29 ± 0.35, respectively, *p* = 0.3) and no difference was evident according to gender lineage (*p* = 0.25). Even the distribution of the pubertal stages in the groups with or without a positive family history was not different taking into account the gender bias (*p* = 0.2 and *p* = 0.8, respectively).

At the onset of disease, hormonal treatment with L-thyroxine was started in 183 of children (47.9%) and the rate was similar in males and females (*p* = 0.8). No significant difference between gender according to the presence of both polyautoimmunity and puberty (*p* = 0.3 and *p* = 0.5, respectively). In our series, all patients were adequately treated, with a good control of signs and symptoms.

### Graves' Disease (GD)

Out of the 382 patients with ATD, 21 subjects were diagnosed with GD, with a higher prevalence in females (*F* = 19, 90.4% vs. M = 2, 9.5%, *p* < 0.001). Mean age at diagnosis was not different in females compared to males (12.14 ± 0.74 vs. 12.76 ± 2.76 years, *p* = 0.6).

Pre-pubertal status was recorded in 19.0% of patients and pubertal condition in 81%, *p* < 0.001, with predominance of late puberty (early puberty 33.0%vs. late puberty in 47.62%, *p* = 0.03); no gender difference between intra- (prepubertal vs. pubertal *p* = 0.8) and inter-puberty groups (prepubertal vs. early pubertal vs. late pubertal, *p* = 0.9) was documented, Figure 1.

Associated autoimmune diseases was detected in 2 males patients (9.52%, *p* < 0.01), that presented both CD and type 1 diabetes, and were at late puberty.

A positive familiarity for autoimmune diseases was reported in 42.86% of patients, without difference in prevalence between males and females (*p* = 0.8). Mean age at onset was not different in subjects with or without a positive family history (12.84±0.70 vs. 11.0±0.50, respectively, *p* = 0.4) and according to the gender lineage (*p* = 0.25). Distribution of the pubertal stages in the groups with or without familiarity was not different according to the gender (*p* = 0.3).

At the onset of disease, metimazole was started in all patients, with a good response in each of them.

## Discussion

Predominance of ATD, both HT and GD, was confirmed in females pediatric and adolescent population. Puberty represents a crucial period for ATD development, without significant gender difference according to pubertal stages. In HT early puberty is a period at greater risk for diagnosis, whereas in GD late puberty is more represented. Poliautoimmunity is more frequent in males compared to females. By contrast, familial predisposition was similarly between genders and into pubertal stages.

The gender differences existing in autoimmune thyroid disease follow a female bias (7) even in a pediatric population, which has displayed a female preponderance of 2 : 1 (3). Different factors may underlie this striking gender difference, including genetic effects, gender differences in sex hormones, immune response and organ vulnerability (7).

Evidence in favor of a genetic basis for the ATD is abundant and the factors that might influence the development of autoimmune disease may be related to genetic susceptibility, chromosomal differences, or epigenetics (12–16).

Genetic studies of patients with autoimmune disorders, including ATD, have shown the role of the major histocompatibility complex (MHC), as compared with other genome areas (17). The association between HLA genes and autoimmune disorders shows a gender bias toward females (17), justifying the predominance of autoimmune diseases, including ATD, in the female population (3, 5, 17, 18).

Findings reported in our series are in line with the evidence that ATD is often accompanied by other organ-specific autoimmune disorders, confirming that common genetic factors may prejudice an individual to developing autoimmunity (7). Although the exact pathogenic mechanism of the coexistence of autoimmune diseases has not been clearly elucidated, the role of HLA haplotypes, such as HLA-B8 and -DR3, in the overlapping of autoimmune disorders was well-supported by higher frequency of these haplotypes in several diseases (19, 20).

We confirmed the higher prevalence of ATD in females. Moreover, we showed that the prevalence of AAD was higher in males compared to females. This result supports that non-HLA genes, such as Polymorphisms in cytotoxic T-lymphocyte anti- gen-4, acid phosphatase locus 1, Discs large homolog 5, interleukin-10, and apolipoprotein E, may be also associated with susceptibility to multiple autoimmune disease (7, 21). Additionally, the stronger susceptibility in women, may support a possible pathogenic role for mechanisms related to genes on the X-chromosome, as reported in animal models (22–25), even though the role of Y chromosome in autoimmune disease and in poliautoimmunity could be not excluded.

The observed familial predisposition to GD and HT confirms the crucial role of the genetic component in the development of this disease. Indeed, we documented that more than 50% of subjects with ATD presented a positive family history, supporting the notion that maternal and paternal inheritance may also contribute to the pathogenesis of autoimmune disease. The lack of the gender specific familial risk suggests that the interaction of genes and environmental factors may underlie parental inheritance of autoimmunity (26).

In adults, a difference between males and females are reported in age at onset of ATD (27). In this study in pediatrics, the gender difference is not evident, but we noted the importance of pubertal stages given the higher prevalence in pubertal compared to prepubertal subjects. These results may confirm that exposure to hormonal changes during puberty plays a fundamental role in immune function (5, 7, 17, 28–30) and consequently in the development of ATD. In our population this is not gender specific, supporting that factors other than hormones, such as environmental effects, could influence the effect of puberty on the disease state (7).

We are aware that there are some limitations in our study starting from recognizing that the retrospective design has numerous disadvantages with an inferior level of evidence. Of note, we did not observe a gender difference in the severity among ATD, as it has been previously reported (7). However, we recorded only the start and the response to treatments, as an index of severity of the disease, and our data do not exclude the effect of the young age on a good prognosis (3, 5, 7). On the other hand, other factors, such as type of symptoms, goiter, ophthalmopathy, hormonal levels would have been necessary to confirm the severity of the disease. Additionally, in this report no environmental factors related to susceptibility to GD and HT, as well as to disease manifestations, have been taken into account (7, 26). Further prospective studies are mandatory to gain a better insight into this research topic.

In conclusion, females are more prone to develop ATD during puberty, in particularly HT in early stage and GD in late stage. The impact of puberty is not different in females and males, supporting the role of additional factors other than sex hormones. We recommend screening for detection of ATD in all children and adolescents with positive family history and other autoimmune diseases, especially in males. Considerations of gender in pediatrics could be important to define pathogenic mechanisms of ATD and to help in early diagnosis and clinical management.

## Data Availability Statement

The datasets generated for this study are available on request to the corresponding author.

## Ethics Statement

The studies involving human participants were reviewed and approved by Ethical Committee of Fondazione IRCCS Policlinico San Matteo, Pavia, Italy. Written informed consent to participate in this study was provided by the participants' legal guardian/next of kids.

## Author Contributions

VC, RN, and DL designed experiments and wrote and supervised the manuscript. CR recorded data of the patients and wrote the manuscript. AI, RAm, FB, AC, and FV recorded data of the patients. AD performed statistical analysis. RAl performed biochemical evaluation. All authors have read and approved the paper.

## Conflict of Interest

The authors declare that the research was conducted in the absence of any commercial or financial relationships that could be construed as a potential conflict of interest.

## References

[1] Ibili ABP Selver Eklioglu B Atabek ME. General properties of autoimmune thyroid diseases and associated morbidities. J Pediatr Endocrinol Metab. (2020) 33:509–15. 10.1515/jpem-2019-0331 32126013

[2] Pasala P Francis GL. Autoimmune thyroid diseases in children. Expert Rev Endocrinol Metab. (2017) 12:129–42. 10.1080/17446651.2017.1300525 30063425

[3] Cappa M Bizzarri C Crea F. Autoimmune thyroid diseases in children. J Thyroid Res. (2010) 2011:675703. 10.4061/2011/675703 21209713 PMC3010678

[4] Minelli R Gaiani F Kayali S Di Mario F Fornaroli F Leandro G. Thyroid and celiac disease in pediatric age: a literature review. Acta Biomed. (2018) 89:11–16. 10.23750/abm.v89i9-S.7872 30561390 PMC6502193

[5] Brown MA Su MA. An inconvenient variable: sex hormones and their impact on T cell responses. J Immunol. (2019) 202:1927–33. 10.4049/jimmunol.1801403 30885988 PMC6425974

[6] Aversa T Corica D Zirilli G Pajno GB Salzano G de Luca F. Phenotypic expression of autoimmunity in children with autoimmune thyroid disorders. Front Endocrinol. (2019) 10:476. 10.3389/fendo.2019.00476 31354636 PMC6640617

[7] Ngo ST Steyn FJ McCombe PA. Gender differences in autoimmune disease. Front Neuroendocrinol. (2014) 35:347–69. 10.1016/j.yfrne.2014.04.004 24793874

[8] Marshall WA Tanner JM Variations in patterns of pubertal changes in boys. Arch Dis Child. (1969) 45:13–23. 10.1136/adc.45.239.13 PMC2020414 5440182

[9] Marshall WA Tanner JM Variations in patterns of pubertal changes in girls. Arch Dis Child. (1969) 44:291–303. 10.1136/adc.44.235.291 5785179 PMC2020314

[10] Calcaterra V Larizza D de Silvestri A Albertini R Vinci F Regalbuto C. Gender-based differences in the clustering of metabolic syndrome factors in children and adolescents. J Pediatr Endocrinol Metab. (2020) 33:279–88. 10.1515/jpem-2019-0134 31927520

[11] Calcaterra V Regalbuto C Dobbiani G Montalbano C Vinci F De Silvestri A. Autoimmune thyroid diseases in children and adolescents with maturity onset diabetes of the young type 2. Horm Res Paediatr. (2019) 92:52–5. 10.1159/000502037 31484194

[12] Shukla SK Singh G Ahmad S Pant P. Infections, genetic and environmental factors in pathogenesis of autoimmune thyroid diseases. Microb Pathog. (2018) 116:279–88. 10.1016/j.micpath.2018.01.004 29325864

[13] Noso S Park C Babaya N Hiromine Y Harada T Ito H. Organ specificity in autoimmune diseases: thyroid and islet autoimmunity in alopecia areata. J Clin Endocrinol Metab. (2015) 100:1976–83. 10.1210/jc.2014-3985 25734250

[14] Jabrocka-Hybel A Skalniak A Piatkowski J Pach D Hubalewska-Dydejczyk A. How far are we from understanding the genetic basis of Hashimoto's thyroiditis? Int Rev Immunol. (2013) 32:337–54. 10.3109/08830185.2012.755175 23617710

[15] Hadj-Kacem H Rebuffat S Mnif-Féki M Belguith-Maalej S Ayadi H Péraldi-Roux S. Autoimmune thyroid diseases: genetic susceptibility of thyroid-specific genes and thyroid autoantigens contributions. Int J Immunogenet. (2009) 36:85–96. 10.1111/j.1744-313X.2009.00830.x 19284442

[16] Jacobson EM Tomer Y. The genetic basis of thyroid autoimmunity. Thyroid. (2007) 17:949–61. 10.1089/thy.2007.0153 17824829

[17] Gough SC Simmonds MJ. The HLA region and autoimmune disease: associations and mechanisms of action. Curr Genomics. (2007) 8:453–65. 10.2174/138920207783591690 19412418 PMC2647156

[18] Selmi C Gershwin ME. Sex and autoimmunity: proposed mechanisms of disease onset and severity. Expert Rev Clin Immunol. (2019) 15:607–15. 10.1080/1744666X.2019.1606714 31033369

[19] Barrett JC Clayton DG Concannon P Akolkar B Cooper JD Erlich HA Type 1 Diabetes Genetics Consortium. Genome-wide association study and meta-analysis find that over 40 loci affect risk of type 1 diabetes. Nat Genet. (2009) 41:703–7. 10.1038/ng.381 19430480 PMC2889014

[20] Zakharova MY Belyanina TA Sokolov AV Kiselev IS Mamedov AE. The contribution of major histocompatibility complex class II genes to an association with autoimmune diseases. Acta Nat. (2019) 11:4–12. 10.32607/20758251-2019-11-4-4-12 31993230 PMC6977962

[21] Du L Yang J Huang J Ma Y Wang H Xiong T. The associations between the polymorphisms in the CTLA-4 gene and the risk of Graves' disease in the Chinese population. BMC Med Genet. (2013) 14:46. 10.1186/1471-2350-14-46 23597029 PMC3637138

[22] Lin Q Hou R Sato A Ohtsuji M Ohtsuji N Nishikawa K. Inhibitory IgG Fc receptor promoter region polymorphism is a key genetic element for murine systemic lupus erythematosus. J Autoimmun. (2010) 34:356–63. 10.1016/j.jaut.2009.08.011 19758787

[23] Santiago-Raber ML Kikuchi S Borel P Uematsu S Akira S Kotzin BL. Evidence for genes in addition to Tlr7 in the Yaa translocation linked with acceleration of systemic lupus erythematosus. J Immunol. (2008) 181:1556–62. 10.4049/jimmunol.181.2.1556 18606711

[24] Subramanian S Tus K Li QZ Wang A Tian XH Zhou J. A Tlr7 translocation accelerates systemic autoimmunity in murine lupus. Proc Nat Acad Sci USA. (2006) 103:9970–5. 10.1073/pnas.0603912103 16777955 PMC1502563

[25] Murphy ED Roths JB. A Y chromosome associated factor in strain BXSB producing accelerated autoimmunity and lymphoproliferation. Arth Rheum. (1979) 22:1188–94. 10.1002/art.178022110 315777

[26] Hoppenbrouwers IA Liu F Aulchenko YS Ebers GC Oostra BA van Duijn CM. Maternal transmission of multiple sclerosis in a dutch population. Arch Neurol. (2008) 65:345–8. 10.1001/archneurol.2007.63 18332246

[27] Manji N Carr-Smith JD Boelaert K Allahabadia A Armitage M Chatterjee VK. Influences of age, gender, smoking, and family history on autoimmune thyroid disease phenotype. J Clin Endocrinol Metab. (2006) 91:4873–80. 10.1210/jc.2006-1402 16968788

[28] Kochummen E Marwa A Umpaichitra V Perez-Colon S Chin VL. Screening for autoimmune thyroiditis and celiac disease in minority children with type 1 diabetes. J Pediatr Endocrinol Metab. (2018) 31:879–85. 10.1515/jpem-2017-0254 29949512

[29] Moulton VR. Sex hormones in acquired immunity and autoimmune disease. Front Immunol. (2018) 9:2279. 10.3389/fimmu.2018.02279 30337927 PMC6180207

[30] Trombetta AC Meroni M Cutolo M. Steroids and autoimmunity. Front Horm Res. (2017) 48:121–32. 10.1159/000452911 28245457

